# The Use of Ultra-Sensitive Molecular Assays in HIV Cure-Related Research

**DOI:** 10.4172/2155-6113.S6-002

**Published:** 2013-05-27

**Authors:** Catherine Kibirige

**Affiliations:** Johns Hopkins Bloomberg School of Public Health, Baltimore, MD, USA

**Keywords:** HIV, Eradication, Nucleic acid tests, PCR, Standardization, Proviral DNA, 2-LTR

## Abstract

Ultra-sensitive laboratory assays based on the Polymerase Chain Reaction (PCR) are playing an increasingly important role in HIV cure-related research. This article reviews the different assays available and how they have evolved. There is a great need for their standardization and for the establishment of reference reagents and testing algorithms to evaluate potential HIV cure-related treatments.

## Introduction

The HIV capsid surrounds two copies of genomic ribonucleic acid (RNA). Replication proceeds with reverse transcription of genomic RNA into a deoxyribonucleic acid (DNA) intermediate. This DNA intermediate is integrated into the host genome where it is referred to as proviral DNA. Unintegrated DNA is present in cells in linear and circular forms. It has a short half-life and disintegrates soon after it is formed [1].

HIV replicates in an error-prone manner that generates a mutation virtually every time the virus replicates. This ongoing mutation allows the emergence of different variants in the host, such as drug-resistant strains or immunological escape mutants. HIV is categorized into HIV type 1 and HIV type 2. HIV-1 is further divided into groups; the major (M) group, the more divergent outlier group (O) group; the non-M, non-O group (N) and the P group. Most HIV infections occur within group M, which is differentiated into subtypes A, B, C, AE, AG, H, J and K. All subtypes and most Circulating Recombinant Forms (CRF's) are found in sub-Saharan Africa. Subtype B is the predominant strain in the US, Europe, Canada and Australia but the prevalence of non-B subtypes in these countries is increasing [1].

The most sensitive FDA approved HIV Nucleic Acid Test (NAT) on the market today is the Abbott Real Time HIV-1 Assay. It has an analytical sensitivity of approximately 25 copies/ml for the 1 ml application. It is approved for the detection of HIV RNA in plasma samples [1]. This assay is not suitable for detecting ultra-low HIV-1 DNA and RNA within host cellular compartments.

Resting memory CD4+ T cells have the ability to harbor latent HIV infection and have been established as an HIV reservoir. The gold standard assay for measuring the frequency of resting memory CD4+ T cells containing latent but replication-competent virus is a viral outgrowth assay that involves harvesting large volumes of blood from an infected patient, sort purifying resting memory CD4+ T cells and activating limiting dilutions of the cells in culture with phytohemagglutinin (PHA). The cells are co-cultured with CD4+ T lymphoblasts from an HIV-negative donor to amplify any virus released from the cells. A p24 Enzyme-Linked Immunosorbent Assay (ELISA) is used to measure infectious units per million cells (IUPM) after two to three weeks of culture. The assay is expensive and labor intensive. It requires large volumes of blood, highly skilled staff and specialized laboratory equipment. The assay has a wide coefficient of variation and cannot be performed with tissue biopsies. The assay may not perform well for eradication approaches that produce only small (1 log) reductions in the size of the latent reservoir [2,3].

There are an increasing number of ultra-sensitive laboratory developed PCR-based assays in use that are capable of detecting lower concentrations of HIV RNA and are capable of detecting HIV DNA. The main advantage of most of the PCR-based assays is that they can be performed on small volumes of fresh and frozen samples including blood and tissue. They are relatively faster and simpler to perform when compared to the gold standard assay. These assays are playing an increasing role in HIV cure-related research. A system needs to be devised for their evaluation and standardization.

Currently, most Taqman PCR assays designed to quantify HIV-1 DNA are optimized for Subtype B and may not be suitable for non-B subtypes. HIV-1 molecular assays do not detect HIV-2. There is a lack of sero-conversion panels for non-B HIV-1 and HIV-2 infections [4].

The most recent ultra-sensitive NATs reported in the literature are addressing this problem by basing oligonucleotide sequences on the Long Terminal Repeat (LTR) region of the HIV genome where sequence conservation across subtypes is at its greatest. Examples include a whole blood leukocyte assay – pbs-rtPCR - that is reported to have 100% sensitivity for 2 input copies of DNA even in the presence of high amounts of genomic DNA (1μg) [5]. Another recently reported assay utilizes a non-traditional 13mer probe with a Locked Nucleic Acid (LNA™) modification. This novel nucleic acid analogue incorporates a 2′-O, 4′-C-methylene bridge that restricts flexibility of the ribofuranose ring and locks it into a rigid C3-endo confirmation (Exiqon). LNA™ bases have improved hybridization affinity and biostability effectively raising the melting temperature of an oligonucleotide by 3 to 8°C for each LNA™ base. This allows for the design of shorter Taqman PCR probes that allow researchers to target very short cross-subtype-conserved sequences within the HIV-1 genome and allows for the development of assays that have broader subtype specificity [4]. Yet another recently reported assay targets the LTR region of the HIV genome and uses a Major Grove Binding (MGB) Probe to achieve greater cross-subtype specificity [6].

## Ultra-Sensitive Molecular Assays in HIV Cure-Related Research

There are currently two general approaches to HIV cure-related research. One approach involves strategies such as very early initiation of HAART and the use of agents that reverse latent infection and thus reduce the latent reservoir. The second approach involves the use of agents that re-activate the latent reservoir and then target and reduce the reactivated virus. The successful outcomes of these potential treatments are classified into “sterilization cures” or “functional cures”.

A sterilization cure occurs when HIV-1 DNA and RNA becomes undetectable in samples from the treated patient when analyzed using ultra-sensitive assays and the patient remains free from disease over a prolonged period without antiretroviral treatment. The first reported case of a sterilization cure from HIV was in 2009 [7,8]. A German HIV positive patient with acute myeloid leukemia received a bone marrow transplant from an HIV negative donor homozygous for a deletion in the CCR5 gene. The patient ceased HIV treatment soon after the procedure. There was a reconstitution and great improvement in the patient's immune profile. He currently remains undetectable for HIV nucleic acids and asymptomatic for HIV-related disease [2,7,8].

A functional cure occurs when low grade HIV-1 DNA and/or RNA is detectable in the treated patient but the patient remains free of HIV-associated disease over a prolonged period of time. Elite controllers of HIV infection are considered prototype cases of a functional cure against HIV [2].

## Ultra-Sensitive Viral Load or Single Copy Assay (SCA)

The Ultra-sensitive viral load assay or SCA was one of the first ultra-sensitive PCR-based viral load assays developed against HIV that is successfully able to detect down to 1 copy of HIV-1 RNA per ml of plasma [9-11]. It has been used extensively to characterize persistent viremia in patients receiving Highly Active Anti-Retroviral Therapy (HAART). The assay requires 7ml of fresh patient plasma, is labor-intensive, and requires highly skilled technicians and specialized laboratory equipment. Some studies have shown a lack of association between residual viremia as detected by the SCA and viral persistence or immune activation [12-14]. It is therefore generally believed that alternative, more reliable methods that do not require large amounts of fresh patient blood and are thus more versatile will have more utility in HIV eradication and other research efforts.

## Total HIV DNA Assays

Assays that measure total (unintegrated and integrated) HIV DNA have been in clinical use for a number of years. Standardized International quality controls are available for them. They quantify replication competent and incompetent virus. They are currently the most feasible tool available for large-scale clinical trials and cohort studies [2,15-20].

Despite a general correlation between total DNA and integrated proviral DNA, there are studies that have shown discordance between the measurements. Some researchers argue that specifically measuring integrated DNA is required as it is a better prognostic marker for treatment failure and success [21].

## Proviral HIV DNA Assays

Measuring proviral DNA in sort purified resting CD4+ T cells or cells derived from other potential HIV reservoirs is considered a marker for latency. Original assays used linker ligation [22], inverse PCR [23] and nested Alu-PCR [24-27]. Various modifications of these assays now exist including more sensitive methods that employ repetitive sampling to maximize on assay sensitivity [28-30].

These assays can be labor intensive. Multiple methods are used in different studies and the reproducibility across multiple laboratories is unknown [2,25,31-33]. Many HIV-1 genomes within cells are defective and as the assays measure both replication competent and incompetent virus, the assays cannot fully ascertain that a viral reservoir is present [3].

## Viral Reactivation Assays

Detection of low grade viral activation will be important for strategies that rely on the reactivation and targeting of previously latent / recently reactivated infected cells [34-36]. Various assays are available that could be used to detect or assess viral reactivation.

### 2-Long Terminal Repeat (LTR) HIV DNA assays

A transient increase in 2-LTR circles in peripheral lymphocytes is interpreted as an indication of ongoing viral replication [2,34,37-39] and could be used to detect recent reactivation. There is, however, considerable controversy as to whether these unintegrated viral DNA forms play a significant role in the HIV replication cycle [29]. There is skepticism about the reliability of the findings as results vary considerably depending on the laboratory, reagents and context under which the assays are performed [40]. The relationship between 2-LTR circles and residual viral replication has, however, been clearly shown in treatment intensification studies using the integrase inhibitor Raltegravir [41]. This treatment intensification results in a specific though transient increase in episomal DNAs in a large number of patients. 2-LTR circle quantification in patients and in vitro models may therefore be a valid marker for virus reactivation and expansion. Standardization of laboratory protocols is required.

### Cell-Associated HIV RNA Assays

These assays measure HIV transcription in productively and latently infected cells. No extracellular HIV RNA is expected in latently infected cells if viral replication has been blocked by HAART or another means. There is a block in nuclear export of multiply spliced RNA and inefficient production of unspliced RNA in these cells. These assays could be used to quantify any increase in transcription following HIV latency reactivation treatment strategies. There are currently very few published studies using these techniques and reproducibility across multiple laboratories is therefore unknown. Assaying for HIV RNA in latent cells may, however, serve as a better marker for reactivation as compared to 2-LTR circles [2,23, 37,42-50].

## Droplet Digital PCR (ddPCR)

Droplet digital PCR (ddPCR) is a nucleic acid detection method that uses the same primers and probes as traditional Taqman RT-PCR. The aqueous reaction mixture is emulsified into thermostable oil and micro-partitioned into picoliter droplets that can contain a single copy or less of target DNA. Following PCR amplification, enumeration of both fluorescing and non-fluorescing droplets allows absolute quantitation of target sequences without relying on the use of standard curves. This greatly reduces the amount of assay “background noise” and allows for greater accuracy and precision. Using 96-well plates, 2 million PCR reactions can be performed simultaneously [51,52]. Emerging data show a significant improvement in precision (∼5 fold decrease in assay coefficient of variation) and a greater than 20 fold accuracy improvement in the detection of 2-LTR circles [52]. Some current limitations include unexplained false positives when used in an endpoint PCR format [52] and a maximum sample input volume of 7.5ul compared to the larger volumes that can be included in real-time PCR [51]. ddPCR therefore requires higher concentrations of DNA substrate in order to maximize sensitivity for detection HIV DNA at very low levels [51]. Partitioning the reactions into droplets may reduce the inhibition usually experienced when large amounts of DNA are used in PCR, but there is a limit to the amount of target DNA copies that can be loaded per droplet without loss of linearity [51]. Overly concentrated samples therefore require dilution for best results. Another limitation of this assay format is that, though it can very accurately detect total DNA, it may not be able to detect integrated HIV-1 DNA as precisely. Current PCR-based methods for specifically detecting integrated HIV require a 2-step amplification protocol, the initial step being, amplification across the HIV DNA and host chromosomal DNA junction. ddPCR could conceivably be used at the second step PCR to quantify first-round amplification products but a standard curve would be required and would thus limit precision [51].

This technology provides a viable alternative to traditional RT-PCR and should be optimized and validated further for use in HIV cure-related research.

## Endpoint Versus Kinetic PCR Assays

Both endpoint and kinetic PCR protocols have been applied to these assays. Generally endpoint PCR is more sensitive but kinetic or quantitative / real-time PCR provides more accurate quantification. Kinetic PCR can control for false positive signals better than endpoint PCR [28].

## Standardizing the Evaluation of HIV Cure-Related Research

PCR-based assays and protocols are allowing for greater and greater sensitivity. Assays can now detect down to 1 input copy of HIV DNA or RNA with greater and greater confidence. The main limitation in sensitivity now lies in sample processing and the number of cell equivalents that can be incorporated into each test, i.e., the number of copies of DNA that can be detected per million cells.

Chun et al. observed a patient with undetectable HIV DNA both in blood and in tissue, but who nonetheless experienced viremia rebound after ART cessation [53]. This shows that undetectable DNA by molecular assays does not rule out infection [34]. Standardized protocols and testing algorithms therefore need to be developed for evaluating HIV cure-related research.

Some of the considerations that will need to be addressed are as follows:

- Sample collecting and handling can greatly affect Nucleic Acid Test Results. It is imperative that specimens be handled, stored and processed correctly and that standardized protocols be devised.
- Specimen concentration and enrichment protocols will need to be utilized to ensure maximum assay sensitivity.
- Researchers need to arrive at a consensus on all the possible reservoir sites within patients and standardize the protocols for sampling and processing cells from these sites.
- Specialized staff training and competency assessment will be required when implementing sample collection and ultrasensitive viral load testing.
- There will be a need for dedicated laboratory equipment with pre and post-amplification areas, reagents and equipment to minimize specimen carry-over and cross-contamination.
- False-positive results and specificity vary depending on the assay used. Low positive results will need to be repeated and assays may need to be used in combination to improve specificity.
- A universal reagent program that provides standardized reagents and panels should be devised.
- A standardized validation protocol needs to be devised for current and future ultra-sensitive laboratory developed assays that are to be used to evaluate potential treatment strategies.
- The HIV strains circulating in the population will need to be considered when selecting the assays
- A standardized protocol for re-testing after a certain number of months or years needs to be devised that incorporates the gold standard assay whenever appropriate and possible.
- Clear definitions of what will be considered a “functional” versus a “sterilization” cure should be devised with wording that takes into account the assay limitations.
- As in clinical diagnosis and monitoring of HIV, different settings may require different assay protocols and algorithms.
